# H_2_O_2_ mediates the crosstalk of brassinosteroid and abscisic acid in tomato responses to heat and oxidative stresses

**DOI:** 10.1093/jxb/eru217

**Published:** 2014-06-04

**Authors:** Jie Zhou, Jian Wang, Xin Li, Xiao-Jian Xia, Yan-Hong Zhou, Kai Shi, Zhixiang Chen, Jing-Quan Yu

**Affiliations:** ^1^Department of Horticulture, Zijingang Campus, Zhejiang University, Yuhangtang Road 866, Hangzhou, 310058, PR China; ^2^Department of Botany & Plant Pathology, Purdue University, West Lafayette, IN 47907-2054, USA; ^3^Key Laboratory of Horticultural Plants Growth, Development and Quality Improvement, Agricultural Ministry of China, Zijingang Road 866, Hangzhou, 310058, PR China

**Keywords:** Abscisic acid, brassinosteroid, hydrogen peroxide, NADPH oxidase, *Solanum lycopersicum*, VIGS.

## Abstract

The production of H_2_O_2_ is critical for brassinosteroid (BR)- and abscisic acid (ABA)-induced stress tolerance in plants. In this study, the relationship between BR and ABA in the induction of H_2_O_2_ production and their roles in response to heat and paraquat (PQ) oxidative stresses were studied in tomato. Both BR and ABA induced increases in *RBOH1* gene expression, NADPH oxidase activity, apoplastic H_2_O_2_ accumulation, and heat and PQ stress tolerance in wild-type plants. BR could only induced transient increases in these responses in the ABA biosynthetic mutant *notabilis* (*not*), whereas ABA induced strong and prolonged increases in these responses in the BR biosynthetic mutant *d* ^*^im*^ compared with wild-type plants. ABA levels were reduced in the BR biosynthetic mutant but could be elevated by exogenous BR. Silencing of *RBOH1* compromised BR-induced apoplastic H_2_O_2_ production, ABA accumulation, and PQ stress responses; however, ABA-induced PQ stress responses were largely unchanged in the *RBOH1*-silenced plants. BR induces stress tolerance involving a positive feedback mechanism in which BR induces a rapid and transient H_2_O_2_ production by NADPH oxidase. The process in turn triggers increased ABA biosynthesis, leading to further increases in H_2_O_2_ production and prolonged stress tolerance. ABA induces H_2_O_2_ production in both the apoplastic and chloroplastic compartments.

## Summary

Brassinosteroids induce H_2_O_2_ accumulation from RBOH1-NADPH oxidase, which first induces ABA biosynthesis and stress tolerance, in turn leading to prolonged H_2_O_2_ production in both apoplast and chloroplast and stress tolerances.

## Introduction

Plants continuously face a myriad of biotic (i.e. fungi, bacteria, viruses, nematodes, and insects) and abiotic (e.g. extreme temperatures, drought, and salt) stresses in the natural environment. To survive such stresses, plants have evolved intricate defence mechanisms to increase their tolerance. Phytohormones, such as auxins, gibberellins, abscisic acid (ABA), cytokinins, salicylic acid, ethylene, jasmonates, brassinosteroids (BRs), and peptide hormones, are all involved in plant adaptation to biotic and abiotic stresses by mediating a wide range of adaptive responses (Lorenzo and Solano, 2005; Mauch-Mani and Mauch, 2005; Rubio *et al.*, 2009; Xia *et al.*, 2009). Moreover, reactive oxygen species (ROS) such as H_2_O_2_ are involved in the regulation of multiple plant responses to a variety of stresses (Neill *et al.*, 2002; Xia *et al.*, 2009; Cui *et al.*, 2011). ROS may function as a second messenger in phytohormone signallings and other important biological processes (Xiong *et al.*, 2002; Chinnusamy and Zhu, 2009; Xia *et al.*, 2009).

BRs are a class of plant steroid hormones involved in a broad spectrum of cellular and physiological processes (Choudhary *et al.*, 2012). In addition to their role in plant growth and development, BRs have been implicated in the regulation of stress responses (Nakashita *et al.*, 2003; Li *et al.*, 2009; Xia *et al.*, 2009). However, the majority of studies on the role of BRs in stress responses rely on exogenous application of BRs or their biosynthesis inhibitor since mutants defective in BRs biosynthesis and signalling often display severely dwarf phenotypes in model plants such as *Arabidopsis* (Nakashita *et al.*, 2003; Liu *et al.*, 2009). These mutants, therefore, may already be under intrinsic cellular stress with altered antioxidant activity (Cao *et al.*, 2005). Accordingly, these mutants are hardly used for studies of the stress responses. As a result, there is a lack of genetic evidence for a role of BRs in plant stress responses. Recently, we found that BRs induce a transient increase in the *Respiratory burst oxidase homolog 1* (*RBOH1*) transcript, NADPH oxidase activity, H_2_O_2_ in the apoplast and nitric oxide (NO). We have shown that BR-induced ROS production is important for BR-induced stress tolerance in cucumber and tomato (Xia *et al*., 2009, 2011; Cui *et al.*, 2011; Nie *et al.*, 2013).

ABA is another important plant hormone with a critical role in the regulation of stress responses (Leung and Giraudat, 1998; Rock, 2000; Shinozaki and Yamaguchi-Shinozaki, 2000). Stresses such as drought induce accumulation of ABA, resulting in increased tolerance (Zhu, 2002). Several lines of evidences show that ABA induces H_2_O_2_ accumulation in the apoplast, which is dependent on NADPH oxidase *RBOH* genes and plays an important role in ABA signalling (Pei *et al.*, 2000; Kwak *et al.*, 2003). Recent studies have demonstrated the interconnections between ABA and other plant hormones such as auxins, cytokinins, gibberellins, ethylene, and BRs in a number of physiological processes (Gazzarrini and McCourt, 2001; Finkelstein *et al.*, 2002; Finkelstein and Gibson, 2002). Several recent studies have also shown that exogenous BRs increased ABA accumulation, and the effect was more significant under stress conditions (Kurepin *et al.*, 2008; Liu *et al.*, 2009; Zhang *et al.*, 2011). Other studies, however, have demonstrated that BRs and ABA displayed an antagonistic relationship in several physiological responses (Zhang *et al.*, 2009). It is not clear whether there is an antagonistic interaction between BRs and ABA in the stress response.

The mechanisms by which BRs enhance plant stress tolerance have so far been largely unknown. Our previous studies have shown that BRs induce ROS production, which is critical for BR-induced stress tolerance in plants (Xia *et al*., 2009, 2011). We have further demonstrated that BRs induce elevated levels of NO, which appear to act downstream of ROS in BR-induced stress tolerance (Cui *et al.*, 2011). Studies using detached shoots and cell suspensions have shown that chemical inhibition of ABA biosynthesis reduced BR-induced stress tolerance (Liu *et al.*, 2009; Zhang *et al.*, 2011), whilst studies in *Arabidopsis* showed that BRs increased the tolerance of mutant plants defective in ABA biosynthesis (Divi *et al.*, 2010). Both ABA and BRs could induce NADPH oxidase *RBOH* genes and increase apoplastic H_2_O_2_ accumulation (Pei *et al.*, 2000; Kwak *et al.*, 2003; Xia *et al.*, 2009). These results present a complex and conflicting picture about the possible relationship between BRs and ABA during the induction of plant stress tolerance. One possible scenario could be that BRs induce the production of ABA, which, in turn, induces apoplastic H_2_O_2_ accumulation and stress tolerance, but, if this is the case, one needs to account for how BRs increase ABA biosynthesis. It is also possible that BRs may themselves induce accumulation of apoplastic H_2_O_2_, which acts to increase biosynthesis of ABA, causing a further increase of H_2_O_2_ production and BR-induced stress responses. To test these possibilities, we compared mutants partially defective in BR biosynthesis with those defective in ABA biosynthesis and *RBOH1*-silenced plants for responses to BRs and ABA through analysis of the changes in stress tolerance, H_2_O_2_ and ABA accumulation, and transcript levels of stress-related genes and antioxidants in tomato.

## Materials and methods

### Plant materials, virus-induced gene silencing (VIGS) construct and *Agrobacterium*-mediated virus infection

Four tomato (*Solanum lycopersicum* L.) genotypes, Condine Red (CR) and its partially BR synthesis mutant *d^* ^*im*^, Ailsa Craig (AC) and its partially ABA-deficient mutant *notabilis* (*not*) were used. Seeds were germinated in a growth medium filled with a mixture of peat and vermiculite (7:3, v/v) in trays in a growth chamber. When the first true leaf was fully expanded, seedlings were transplanted into plastic pots (15cm diameter×15cm deep, one seedling per pot) containing the same medium and were watered daily with Hoagland nutrient solution. The growth conditions were as follows: a 14h photoperiod, temperature of 25/20 °C (day/night), and photosynthetic photon flux density (PPFD) of 600 µmol m^–2^ s^–1^.

The tobacco rattle virus (TRV) VIGS construct used for the silencing of the tomato *RBOH1* gene were generated by cloning a 311bp *RBOH1* cDNA fragment, which was PCR amplified using the forward primer (5′-ATACGCGAGCTCAAGAATGGGGTTGATATTGT-3′) and the reverse primer (5′-ATACCGCTCGAGCTCTGACTTATT CCTTAC-3′) according to Liu *et al.* (2002). The amplified fragment was digested with *Sac*I and *Xho*I and ligated into the same sites of pTRV2. The resulting plasmids were transformed into *Agrobacterium tumefaciens* GV3101. *Agrobacterium*-mediated virus infection was performed as described previously (Ekengren *et al.*, 2003). Plants were then kept at 23/21 °C under 120 µmol m^–2^ s^–1^ PPFD for 30 d before they were used for experiments (Kandoth *et al.*, 2007).

### Stress treatments and analysis of chlorophyll fluorescence

To evaluate BR-induced tolerance to various stresses, tomato seedlings at the five-leaf stage were sprayed with 24-epibrassinolide (EBR) at 0, 30, 100, 200, 500, and 1000nM or with ABA at 0, 10, 20, 50, 100, and 200 μM. After 3 or 24h, the seedlings were exposed to heat-shock stress or paraquat (PQ) stress, respectively. For the heat-shock stress, the tomato seedlings were maintained at 42 °C under 800 µmol m^–2^ s^–1^ PPFD for 6h. For the PQ stress, the seedlings were sprayed with 20 μM PQ and subsequently maintained at 600 µmol m^–2^ s^–1^ PPFD and 25 °C for 3h. To determine the role of ROS from different sources, the plants were pre-treated with water, 50 µM diphenylene iodonium (DPI, an NADPH oxidase inhibitor) or 5mM dimethylthiourea (DMTU, an H_2_O_2_ scavenger) for 12h and subsequently treated with 200nM EBR or 50 µM ABA. At the end of each experiment, the fourth leaf from the bottom was used for biochemical analysis.

Chlorophyll fluorescence was measured using an Imaging-PAM Chlorophyll Fluorometer equipped with a computer-operated PAM-control unit (IMAG-MAXI; Heinz Walz, Effeltrich, Germany). The seedlings were maintained in the dark for more than 30min before the measurements were performed. The intensities of the actinic light and saturating light were 280 and 4000 μmol mol^–2^ s^–1^ PPFD, respectively. The maximum quantum yield of photosystem II (Fv/Fm) was measured and calculated according to van Kooten and Snel (1990). There were three replicates for each treatment and each replicate had 12 plants.

### H_2_O_2_ quantification, histochemical analysis, and cytochemical detection

H_2_O_2_ was extracted from leaf tissue according to Doulis *et al.* (1997) and measured as described in our earlier study (Xia *et al.*, 2011). Histochemical staining of H_2_O_2_ in the plants was detected as described previously (Thordal-Christensen *et al.*, 1997). Leaves from tomato plants treated with water, EBR, or ABA were detached and placed in a solution containing 1mg ml^–1^ of 3,3′-diaminobenzidine (DAB, pH 5.5) for 4h after a brief vacuum infiltration. The leaf discs were boiled in 90% (v/v) ethanol for 10min, stored in 50% glycerol, and photographed (BX61; Olympus Co., Tokyo, Japan). H_2_O_2_ was also visualized at the subcellular level using CeCl_3_ for localization, as described previously (Bestwick *et al.*, 1997; Zhou *et al.*, 2012). The sections were examined using a transmission electron microscope (H7650; Hitachi, Tokyo, Japan) at an accelerating voltage of 75kV to detect the electron-dense CeCl_3_ deposits that were formed in the presence of H_2_O_2_.

### NADPH oxidase and antioxidant analysis

For the determination of NADPH oxidase activity, leaf plasma membranes were isolated using a two-phase aqueous polymer partition system (Larsson *et al.*, 1987). The NADPH -dependent O_2_^•–^-generating (EC 1.6.3.1) activity in isolated plasma membrane vesicles was determined as described previously (Xia *et al.*, 2009; Zhou *et al.*, 2012). The rates of O_2_^•–^ generation were calculated using an extinction coefficient of 21.6mM^–1^ cm^–1^. Reduced (GSH) and oxidized (GSSG) glutathione were determined according to Rao and Ormrod (1995). Reduced (AsA) and oxidized (DHA) ascorbate was measured following the method of Tamura and Suzuki (1991).

For the antioxidant enzyme assays, leaf tissue (0.3g) was ground with 2ml of ice-cold buffer containing 50mM PBS (pH 7.8), 0.2mM EDTA, 2mM AsA, and 2% (w/v) polyvinylpolypyrrolidone. The homogenates were centrifuged at 12 000*g* for 20min, and the resulting supernatants were used for the determination of enzyme activity. All steps were performed at 4 °C. The protein content was determined following the method of Bradford (1976) using bovine serum albumin as a standard. Superoxide dismutase (SOD) activity was assayed by the photochemical method described by Stewart and Bewley (1980). Ascorbate peroxidase (APX) was assayed according to Nakano and Asada (1981). The activity of catalase (CAT) was measured using the method of Cakmak and Marschner (1992). Glutathione reductase (GR) activity was measured following the protocol of Foyer and Halliwell (1976). All the spectrophotometric analyses were performed using a SHIMADZU UV-2410PC spectrophotometer (Shimadzu Co., Kyoto, Japan).

### Measurement of endogenous ABA levels

For ABA extraction, 1g of fresh leaves was ground in a mortar and homogenized in extraction solution (80% methanol, v/v). The extracts were centrifuged at 10 000*g* for 20min. The supernatant was eluted through a Sep-Pak C18 cartridge (Waters, Milford, MA, USA) to remove the polar compounds and subsequently stored at –20 °C for an ELISA. The ELISA procedures were conducted following the manufacturer’s instructions (China Agricultural University, Beijing, China). ABA was determined using the Multimode Plate Reader Label-free System (PerkinElmer, Boston, MA, USA).

### Total RNA isolation and quantitative real-time PCR (qRT-PCR) analysis

Total RNA was isolated from tomato leaves using Trizol reagent (Sangon, Shanghai, China), according to the manufacturer’s recommendations. Genomic DNA was removed with am RNeasy Mini Kit (Qiagen, Hilden, Germany). Total RNA (1 µg) was reverse transcribed using a ReverTra Ace qPCR RT kit (Toyobo, Osaka, Japan) following the manufacturer’s instructions. Gene-specific RT-PCR primers were designed based on their cDNA sequences (Supplementary Table S1 at *JXB* online). Two of these genes encoded transcription factors (*WRKY1* and *WRKY72*), and six were involved in stress responses: *MAPK1* (encoding mitogen-activated protein kinase 1), *HSP70* (encoding a 70kDa heat-shock protein), *Cu/Zn-SOD* (encoding Cu/Zn-SOD), *cAPX* (encoding cytosolic ascorbate peroxidase), *CAT1* (encoding catalase 1), and *GR1* (encoding glutathione reductase 1).

qRT-PCR was performed using a iCycleri Q^TM^ real-time PCR detection system (Bio-Rad, Hercules, CA, USA). Each reaction (25 µl) consisted of 12.5 µl of SYBR Green PCR Master Mix (Takara, Chiga, Japan), 1 µl of diluted cDNA and 0.1 µM forward and reserve primers. The PCR cycling conditions were as follows: 95 °C for 3min, followed by 40 cycles of 95 °C for 10 s and 58 °C for 45 s. The tomato *Actin* gene was used as an internal control. Relative gene expression was calculated according to Livak and Schmittgen (2001).

### Statistical analysis

The experimental design was a completely randomized block design with three replicates. Statistical analysis of the bioassays was performed using the SAS statistical package. The differences between the treatment means were separated by Tukey’s test at a level of *P*<0.05.

## Results

### Dynamic dependency of ABA biosynthesis in BR-induced tolerance

**Fig. 1.**
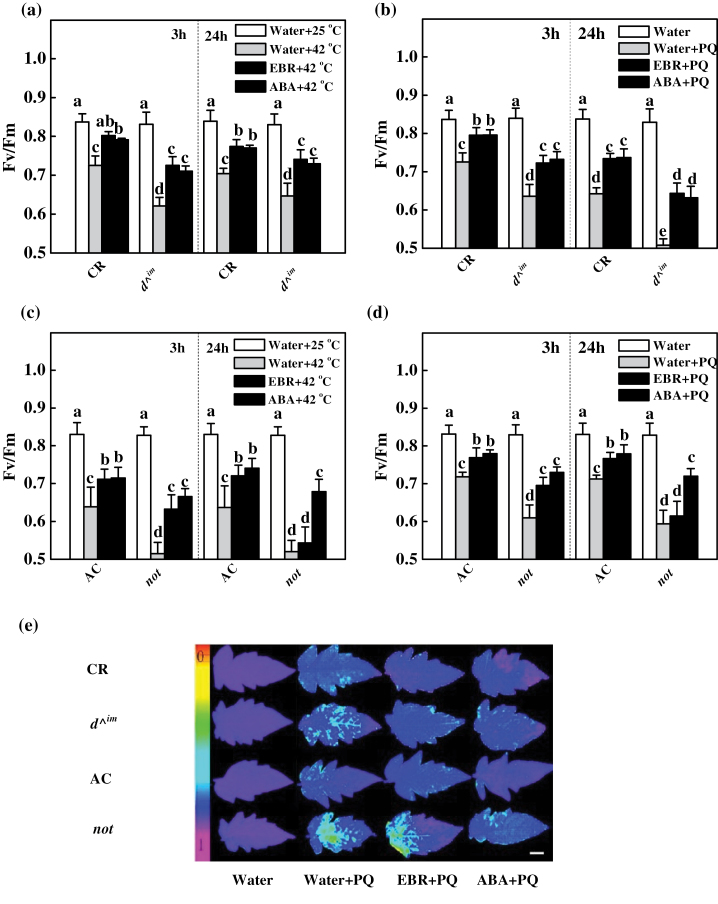
Effects of BR and ABA levels on heat-shock and photo-oxidative stress tolerances in BR- and ABA-deficient plants. (a, c) Fv/Fm values of plants after exposure to a 6h heat shock (42 °C under 800 µmol m^–2^ s^–1^). (b, d) Fv/Fm values of plants after exposure to 3h of photo-oxidative stress (20 μM PQ). For (a)–(d), EBR (200nM) or ABA (50 μM) was applied at 3 and 24h before the plants were exposed to the heat-shock and PQ stresses, respectively. (e) Images of the Fv/Fm of leaves challenged with 20 μM PQ and pre-treated with EBR (200nM) or ABA (50 μM) for 24h. The false colour code depicted at the left of the image ranges from 0 (black) to 1 (purple). Bar, 1.0cm. Twelve plants were used for each treatment, and Fv/Fm values were determined with the entire fourth leaf as the area of interest. The data are means of 12 replicate plants (±SD). Means denoted by the same letter do not differ significantly at *P≤*0.05 according to Turkey’s test.

To study the relationship between ABA- and BR-induced stress tolerance, we compared BR-deficient *d^* ^*im*^ and ABA-deficient *not* mutants and their corresponding wild-type plants to determine their EBR- and ABA-induced tolerance to heat stress and PQ oxidative stress, respectively. Without heat or PQ treatment, all mutant and wild-type plants had similar Fv/Fm values (close to 0.83). Exposure to heat (Fig. 1a, c) and PQ (Fig. 1b, d) resulted in significant reductions in Fv/Fm at 6 and 3h, respectively, and this decrease was more significant in the *d^* ^*im*^ and *not* plants, suggesting that a defect in either BR or ABA accumulation reduced tolerance to heat and photo-oxidative stresses. Pre-treatment with EBR or ABA at 3 or 24h prior to the exposure to heat and PQ significantly increased Fv/Fm in both wild-type plants. However, although ABA restored heat and PQ tolerance in the BR-deficient *d^* ^*im*^ mutant plants, EBR restored the heat and PQ tolerance of the *not* plants at 3h but was ineffective at 24h after its application (Fig. 1). We also analysed the changes in electrolyte leakage after exposure to heat-shock and photo-oxidative stress and similar results were observed (Supplementary Fig. S1 at *JXB* online). Thus, ABA was able to rescue the BR-deficient *d^* ^*im*^ mutant plants, but EBR could only partially and transiently rescue the defective stress tolerance of the ABA-deficient *not* mutant. This observation suggested that ABA might act downstream of BRs in plant stress responses. Furthermore, ABA-induced PQ tolerance was effectively blocked by pre-treatment with DMTU but not by DPI, whilst EBR-induced PQ tolerance in CR was abolished by both DPI and DMTU (Supplementary Fig. S2 at *JXB* online). These results suggested that non-apoplastic ROS were also responsible for the observed ABA-induced PQ tolerance.

### Kinetics of EBR- and ABA-induced *RBOH1* expression and H_2_O_2_ accumulation

**Fig. 2.**
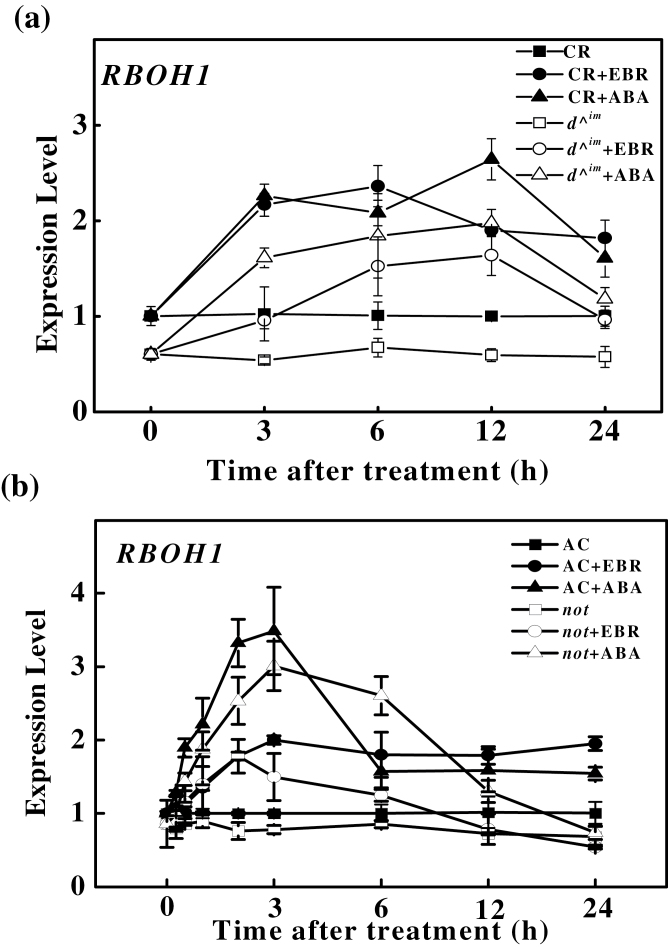
Influence of EBR and ABA treatments on the time dependence of *RBOH1* transcription. EBR and ABA were foliarly applied at concentrations of 200nM and 50 μM, respectively. The data are means of three replicates (±SD). Means denoted by the same letter do not significantly differ at *P≤*0.05 according to Turkey’s test.

*RBOH1*-NADPH oxidase plays an important role in BR- and ABA-induced stress responses in plants (Kwak *et al.*, 2003; Xia *et al.*, 2009). To determine whether the ABA dependence of BR-induced tolerance was related to the *RBOH1* transcript, which is important in stress responses (Zhou *et al.*, 2012), we compared the BR-deficient *d^* ^*im*^ and ABA-deficient *not* mutants and their respective wild-type counterparts to determine EBR- and ABA-induced *RBOH1* transcription. *RBOH1* transcript levels were increased as early as 3h and remained elevated up to 24h after EBR or ABA treatment in CR and the BR-deficient *d* ^*^im*^ mutant (Fig. 2a). *RBOH1* was also rapidly elevated from 0.5 to 24h in AC after ABA or EBR treatment (Fig. 2b). ABA treatment increased the transcript levels of *RBOH1* in the ABA-deficient *not* mutant plants almost as rapidly and as strongly as in the wild-type plants (Fig. 2b). In contrast, EBR induced only a small and transient elevation of *RBOH1* transcripts at early time points after treatment in the *not* mutant. Thus, EBR is capable of inducing *RBOH1* expression; however, strong and sustained induction of the gene by EBR is dependent on a sufficient ABA level.

**Fig. 3.**
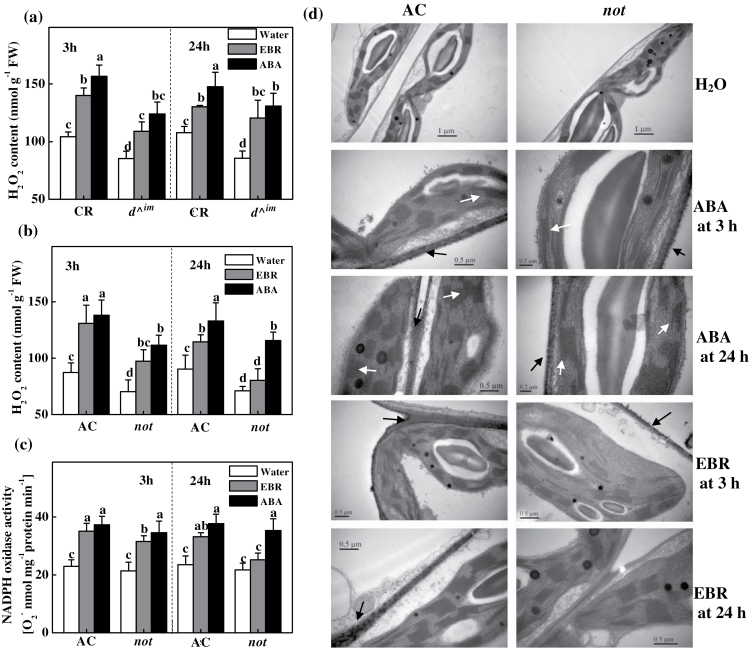
Influence of EBR and ABA treatments on the time dependence of H_2_O_2_ accumulation and NADPH oxidase activity. (a, b) H_2_O_2_ accumulation. (c) NADPH oxidase activity. (d) Cytochemical detection of H_2_O_2_. Black arrows indicate membrane H_2_O_2_ accumulation and white arrows indicate CeCl_3_ precipitates in the chloroplasts. EBR and ABA were applied foliarly at concentrations of 200nM and 50 μM, and samples were taken at 3 and 24h after the treatments, respectively. The data are means of three replicates (±SD). Means denoted by the same letter do not differ significantly at *P≤*0.05 according to Turkey’s test. FW, Fresh weight.

A quantitative analysis of the H_2_O_2_ content supported the observation that EBR was almost as effective as ABA at inducing H_2_O_2_ in wild-type and BR-deficient *d* ^*^im*^ mutant plants, whilst it induced H_2_O_2_ accumulation only at 3h and not at 24h in the ABA-deficient *not* mutant plants (Fig. 3a, b). Similarly, both EBR and ABA increased NADPH oxidase activity in the wild-type plants at 3 and 24h, whilst EBR increased NADPH oxidase activity only at 3h and not at 24h in the *not* plants (Fig. 3c).

*In situ* DAB staining also revealed increased accumulation of H_2_O_2_ in the EBR- and ABA-treated wild-type plants (Supplementary Fig. S3 at *JXB* online). H_2_O_2_ accumulation was observed in the chloroplasts in the ABA-treated leaves but not in the EBR-treated leaves. Pre-treatment with DPI and DMTU abolished the EBR-induced H_2_O_2_ accumulation, whereas only DMTU could abolish the ABA-induced H_2_O_2_ accumulation (Supplementary Fig. S3b). Using CeCl_3_-based procedures, we further observed increased H_2_O_2_ accumulation in the apoplast and chloroplasts in response to ABA in the AC plants at 3 and 24h (Fig. 3d). In comparison, increased H_2_O_2_ accumulation was observed only in the apoplast in the EBR-treated AC plants. Again, ABA induced H_2_O_2_ accumulation in the apoplast and chloroplasts in the *not* plants; in sharp contrast, EBR induced H_2_O_2_ accumulation in the apoplast but not in the chloroplasts at 3h (Fig. 3d). However, no such apoplastic H_2_O_2_ accumulation was observed in the EBR-treated *not* plants at 24h. Thus, it is plausible that ABA is able to activate another ROS production pathway that is not dependent on *RBOH1*-NADPH oxidase.

### BR and ABA differentially trigger H_2_O_2_ accumulation in tomato plants

**Fig. 4.**
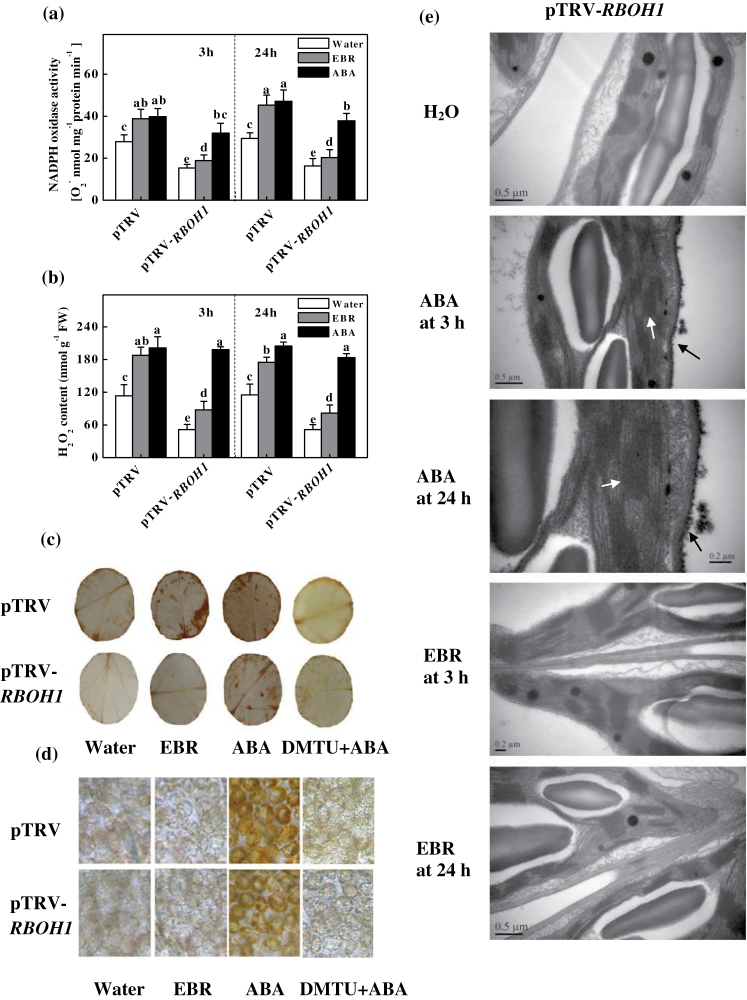
EBR- and ABA-induced H_2_O_2_ and NADPH oxidase activity is dependent on expression of *RBOH1*. (a) NADPH oxidase activity. (b) H_2_O_2_ accumulation. (c, d) *In situ* detection of H_2_O_2_ in leaves. The plants were pre-treated with 5mM DMTU or water for 12h and subsequently treated with 200nM EBR or 50 µM ABA. After 24h, DAB staining of leaf discs was performed. H_2_O_2_ accumulation was detected using an Olympus motorized system microscope (BX61; Olympus, Tokyo, Japan) at 2× (c) and 400× (d) magnification. (e) Cytochemical detection of H_2_O_2_. Black arrows indicate H_2_O_2_ on the membranes and white arrows indicate CeCl_3_ precipitates in the chloroplasts.

To determine the role of *RBOH1* in BR- and ABA-induced H_2_O_2_ accumulation in the apoplast, we silenced *RBOH1* using VIGS. As observed in our previous study (Nie *et al.*, 2013), silencing of *RBOH1* resulted in a 70–80% decrease in *RBOH1* transcript levels in the leaves (data not shown). Importantly, EBR-induced NADPH oxidase activity was compromised in the *RBOH1*-silenced (pTRV-*RBOH1*) plants at both 3 and 24h after EBR treatment (Fig. 4a). In comparison, ABA was also effective in inducing NADPH oxidase activity in pTRV-*RBOH1* plants, although the increase was less significant compared with that observed in the non-silenced plants (pTRV) (Fig. 4a). At 3 and 24h after EBR treatment, both the basal and EBR-induced H_2_O_2_ levels were significantly lower in the pTRV-*RBOH1* than in the pTRV control plants (Fig. 4b). Additionally, ABA induced H_2_O_2_ production at 3 and 24h in both pTRV control plants and pTRV-*RBOH1* plants. However, EBR failed to significantly increase H_2_O_2_ staining in the *RBOH1-*silenced plants, whereas ABA substantially increased H_2_O_2_ staining (Fig. 3c).

It is possible that ABA is able to activate another ROS production pathway that is not dependent on *RBOH1*-NADPH oxidase. To confirm this hypothesis, we detected H_2_O_2_ accumulation in the chloroplasts and leaf discs of the *RBOH1*-silenced plants. Strong H_2_O_2_ accumulation was observed in the apoplast and chloroplasts of control and *RBOH1*-silenced leaves at 3 and 24h after ABA treatment (Fig. 4e), and this accumulation could be abolished by DMTU pre-treatment (Fig. 4c, d). In contrast, no such H_2_O_2_ accumulation was observed in the chloroplasts of EBR-treated plants (Fig. 4d, e), and, importantly, EBR failed to induce H_2_O_2_ accumulation in the apoplast of *RBOH1*-silenced plants (Fig 4c–e). These results suggested that, although EBR increased H_2_O_2_ accumulation almost exclusively in the apoplast through an *RBOH1*-dependent mechanism, ABA induced an additional ROS production pathway in the chloroplasts that was not dependent on NADPH oxidase in the apoplast. Our results also revealed that the EBR-induced sustained accumulation of H_2_O_2_ was largely dependent on ABA synthesis.

### BR and ABA induce tolerance against photo-oxidative stress via different ROS generation pathways

**Fig. 5.**
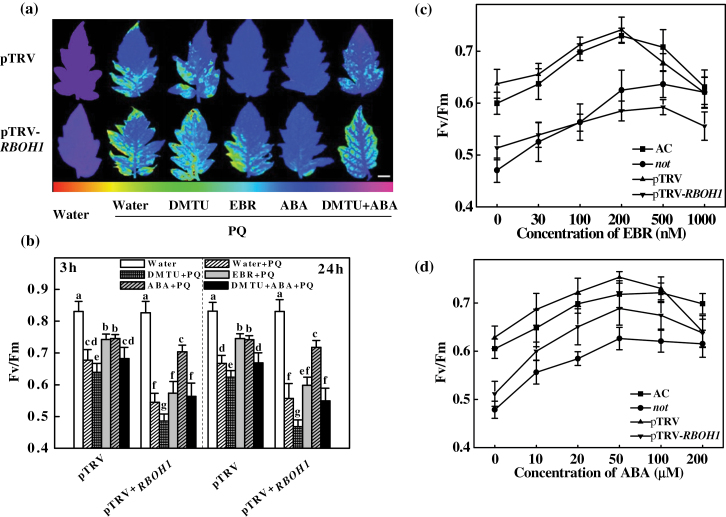
*RBOH1*-dependent EBR- and ABA-induced tolerance and dose–response curves illustrating the effects of EBR and ABA on Fv/Fm under photo-oxidative stress (20 μM PQ). (a, b) Images and maximum photosystem II quantum yield (Fv/Fm) of PQ-challenged leaves pre-treated with EBR, ABA, and DMTU. The false colour code depicted below the image ranges from 0 (black) to 1 (purple). Bar, 1.0cm. The plants were pre-treated with water or 5mM DMTU for 12h and subsequently treated with 200nM EBR or 50 µM ABA. After 24h, the plants were challenged with 20 µM PQ for 3h at 600 µmol m^–2^ s^–1^ light intensity and 25 °C. (c, d) Fv/Fm values of plants exposed to 3h of 20 μM PQ stress at 3h after treatment with different concentrations of EBR and ABA. Fv/Fm was determined using the entire leaf as the area of interest. Before PQ stress was applied, Fv/Fm was 0.827 for AC plants, 0.823 for *not* plants, 0.830 for pTRV plants, and 0.828 for pTRV-*RBOH1* plants. The data are means of 12 replicate plants (±SD).

To determine the role of *RBOH1* in BR- and ABA-induced stress tolerance, pTRV and pTRV-*RBOH1* plants were exposed to PQ at 3 and 24h after EBR or ABA treatment, respectively. EBR treatment alleviated the PQ-induced decrease in Fv/Fm in the pTRV control plants but not in the pTRV-*RBOH1* plants. In comparison, ABA alleviated the Fv/Fm reduction in both the pTRV and pTRV-*RBOH1* plants (Fig. 5a, b). The ABA-induced PQ tolerance was compromised by pre-treatment with DMTU (Fig. 5a, b).

We further analysed the effects of different concentrations of EBR and ABA on the tolerance to photo-oxidative stress at 3h (Fig. 5c, d). EBR treatment increased Fv/Fm only marginally at a very low concentration (30nM), whereas EBR increased Fv/Fm by approximately 20% at a higher concentration (200nM) when compared with the untreated control in AC and pTRV plants (Fig. 5c). However, further increases in the EBR concentration resulted in a reduction in its beneficial effects. The most effective concentration of EBR was approximately 200nM in the AC and pTRV plants; however, in ABA-deficient *not* plants, a higher EBR concentration (500nM) was required to reach its maximum effects. Furthermore, the pTRV-*RBOH1* plants were not responsive to EBR at any of the concentrations tested. Similarly, the extent of ABA-induced tolerance was dependent on the applied concentration, although ABA also induced tolerance in the pTRV-*RBOH1* plants (Fig. 5d). Thus, ABA-induced stress tolerance is independent of *RBOH1*-NADPH oxidase.

### Relationships among ABA biosynthesis, BR biosynthesis, and BR-induced apoplastic H_2_O_2_

**Fig. 6.**
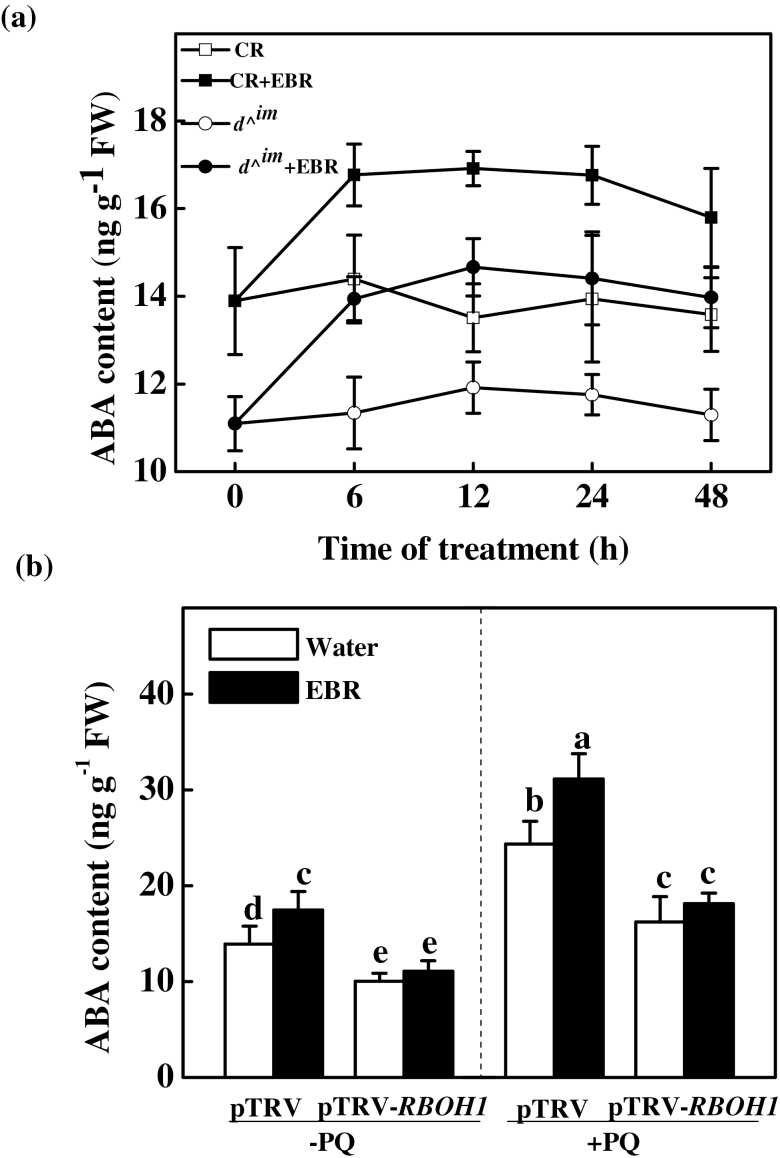
Influence of BR level on ABA biosynthesis and *RBOH1* silencing. (a) Time course of EBR-induced ABA accumulation in plants with different BR levels. The plants were treated with 200nM EBR and samples were collected at the time points indicated. (b) Effects of *RBOH1* silencing and PQ treatment on ABA accumulation. pTRV and pTRV-*RBOH1* plants were treated with water or 200nM EBR for 12h and subsequently exposed or not to 20 µM PQ stress for 12h. The data are means of three replicates (±SD). Means denoted by the same letter do not differ significantly at *P≤*0.05 according to Turkey’s test.

To determine whether EBR treatment increases ABA biosynthesis and, if so, whether EBR-induced H_2_O_2_ accumulation in the apoplast is required for increased ABA accumulation, we analysed the response of ABA to EBR and PQ in wild-type, *d^* ^*im*^, and pTRV-*RBOH1* plants (Fig. 6). Interestingly, compared with the CR control plants, *d^* ^*im*^ plants exhibited reduced ABA contents. In both the CR and *d^* ^*im*^ plants, ABA accumulation was significantly increased after EBR treatment for the duration of the experiment (Fig. 6a). Similarly, EBR induced ABA accumulation in the pTRV plants, and this increase was even higher after exposure to PQ stress (Fig. 6b). However, the ABA concentration in the pTRV-*RBOH1* plants was increased only after PQ treatment and not after EBR treatment. These results strongly suggested that EBR treatment induced ABA biosynthesis, particularly under stress conditions, and that this BR-induced ABA biosynthesis was dependent on *RBOH1*.

### Dynamics of defence-related gene transcription under BR or ABA treatment

**Fig. 7.**
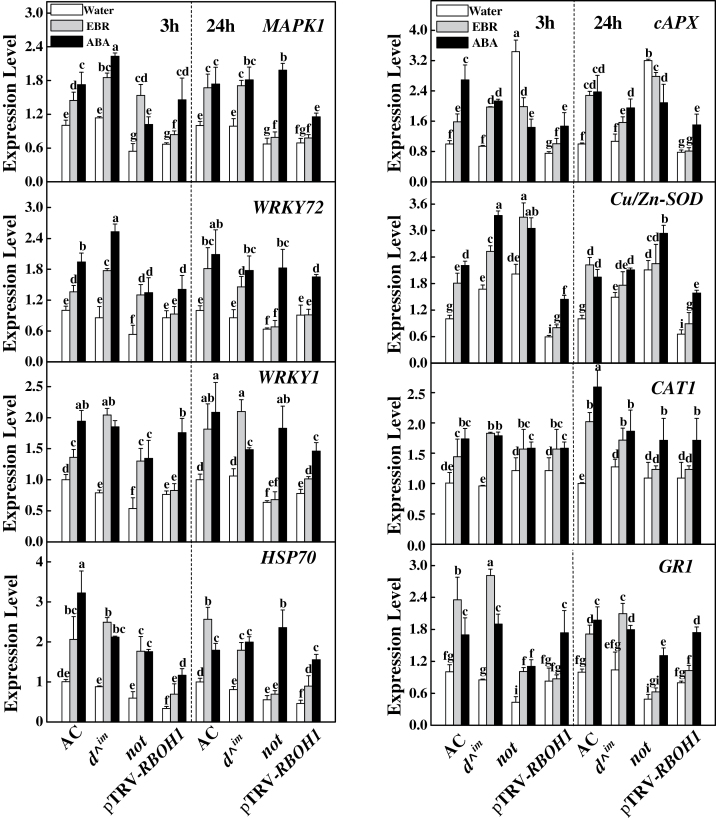
Effects of EBR and ABA treatments on the expression of stress-responsive genes. Leaf samples were collected at 3 and 24h after foliar treatment with EBR (200nM) or ABA (50 μM). qRT-PCR analysis was performed to examine the steady-state levels of mRNAs in the plants. The data are means of three replicates (±SD). Means denoted by the same letter do not differ significantly at *P≤*0.05 according to Turkey’s test. The expression level of genes in CR and pTRV plants was set to 1 for comparative expression analysis of the same genes in the *not* and pTRV-*RBOH1* plants, respectively.

To analyse further the underlying molecular mechanisms of BR- and ABA-induced stress tolerance, we analysed the changes in defence-related genes at 3 and 24h after EBR or ABA treatment (Fig. 7). Two of these genes are involved in gene transcription (*WRKY1* and *WRKY72*) and have been identified as transcriptional regulators involved in defence stress responses (Bhattarai *et al.*, 2010; Molan and El-Komy, 2010), and six are involved in stress responses (*MAPK1*, *HSP70*, *Cu/Zn-SOD*, *cAPX*, *CAT1*, and *GR1*). As shown in Fig. 7, there was no significant difference in the expression of these genes between the wild-type and *d* ^*^im*^ plants, with the exception of *Cu/Zn-SOD*, which was expressed at higher levels in the *d* ^*^im*^ plants than in the wild-type plants. In contrast, the transcript levels of *MAPK1*, *WRKY72*, *WRKY1*, *HSP70*, and *GR1* were decreased and *cAPX* and *Cu/Zn-SOD* transcripts were hyper-accumulated in the ABA *not* mutant when compared with the corresponding wild-type line. The expression levels of these genes did not differ in the pTRV-*RBOH1* plants compared with the pTRV plants with the exception of *HSP70*, which was downregulated in the pTRV-*RBOH1* plants.

EBR or ABA treatment increased the transcription of defence-related genes in the AC plants at 3 and 24h. In the *not* plants, the transcription of these genes was also increased by ABA; however, EBR increased their transcription only at 3h and not at 24h. *cAPX* was downregulated by both BR and ABA in the *not* plants. In the pTRV-*RBOH1* plants, these defence-related genes were induced by ABA but not by EBR at 3 and 24h.

### Interplay of BR and ABA in the regulation of cellular redox homeostasis

**Fig. 8.**
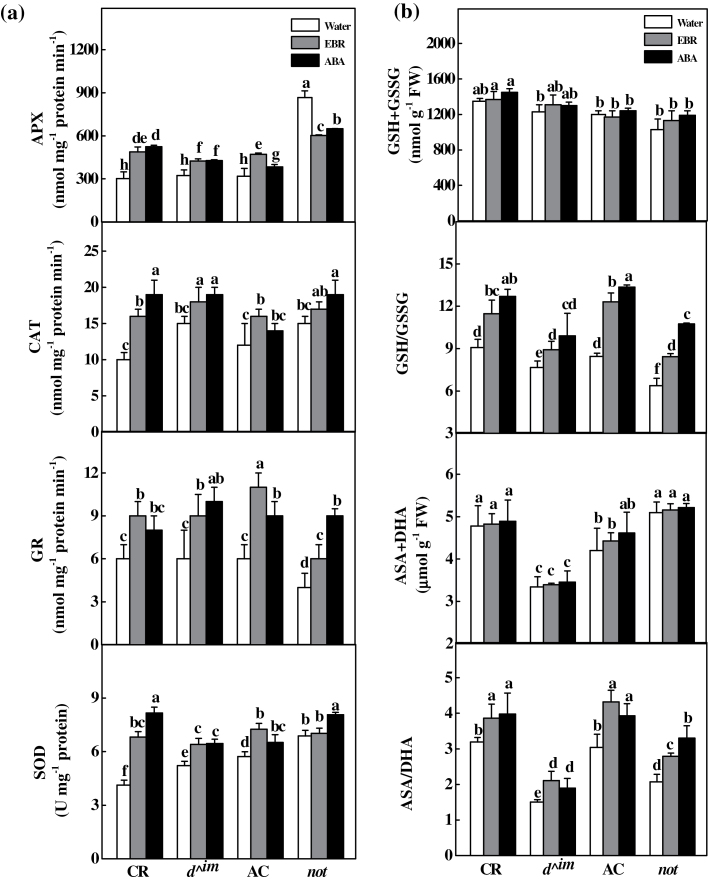
Changes in antioxidant enzymes, glutathione and ascorbic acid redox homeostasis after EBR or ABA treatment. Tomato plants with different levels of endogenous BRs and ABA were treated with 200nM EBR or 50 µM ABA and the activities of antioxidant enzymes and glutathione redox homeostasis were determined 24h later. The values are means of three replicates (±SD). Means denoted by the same letter do not differ significantly at *P≤*0.05 according to Turkey’s test.

The activities of the antioxidant enzymes SOD, CAT, APX, and GR were increased at 24h in the two wild-types and the *d* ^*^im*^ mutant after EBR or ABA treatment (Fig. 8a). In the *not* plants, ABA, but not EBR, increased the activities of SOD, CAT, and GR at 24h after treatment. In comparison, APX activity was not affected by EBR treatment, but was decreased after ABA treatment. The total glutathione and ascorbate contents were not altered at 24h after EBR or ABA treatment, although the ratios of GSH/GSSG and AsA/DHA were increased by the EBR or ABA treatment (Fig. 8b). Therefore, exogenous BRs induced plant responses to oxidative stress in an ABA-dependent manner at 24h after treatment.

## Discussion

**Fig. 9.**
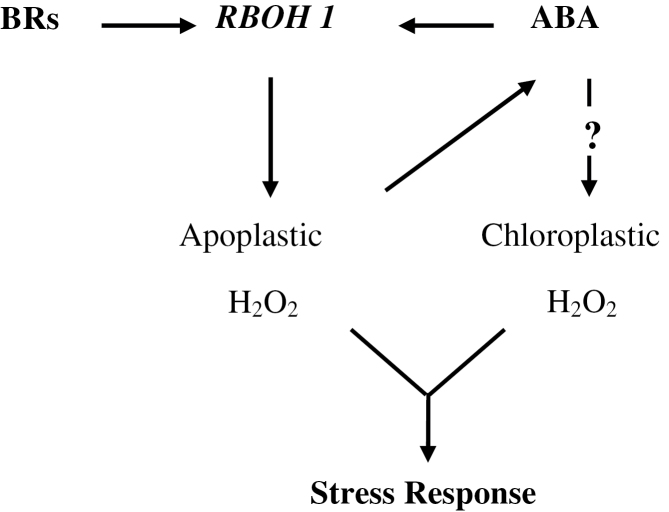
A proposed model for the induction of stress tolerance by BRs and ABA in tomato. Upon perception of a BR signal, plasma membrane-bound *RBOH1*-NADPH oxidase induces the production of apoplastic H_2_O_2_, which first induces the biosynthesis of ABA, in turn leading to a further increase in H_2_O_2_ production in both the apoplastic and chloroplastic compartments. Consequently, prolonged stress tolerance is induced.

An increasing body of evidence supports the role of BRs in stress and defence responses. In this study, we have presented evidence that BR-induced stress tolerance involves a positive feedback mechanism in which BRs induce a rapid and transient H_2_O_2_ production by NADPH oxidase, which first triggers increases in ABA biosynthesis that lead to a further increase in H_2_O_2_ production. Consequently, prolonged stress tolerance is induced (Fig. 9).

### BR-induced stress tolerance is associated with changes in the cellular redox state

We demonstrated that endogenous BR levels are closely related to stress tolerance (Fig. 1). Intriguingly, reduced BR levels in the BR biosynthetic mutant plants were associated with reduced ratios of GSH/GSSG and AsA/DHA, although exogenously applied BRs significantly increased the transcript levels of almost all defence-related genes, the activity of antioxidant enzymes, and the GSH/GSSG and AsA/DHA ratios (Fig. 8b). Several recent studies have revealed that stress-inducible genes are constitutively upregulated in the BR-insensitive *Arabidopsis* mutant *bri1* and that heat-shock-induced oxidative stress depends on BR levels in tomato (Kim *et al.*, 2010; Mazorra *et al.*, 2011). In the present study, we observed increases in *Cu/Zn-SOD* expression and SOD activity in *d* ^*^im*^ plants, suggesting that BR deficiency may induce partial oxidative stress in plants. In many cases, maintaining a reduced cellular redox state is important for plant growth and tolerance to biotic and abiotic stresses. Indeed, during the development of systemic acquired resistance, there is an initial ROS accumulation that perturbs the cellular redox state, which in turn activates the NPR1 pathway to activate the *PR1* gene (Mou *et al.*, 2003). Most recently, we found that BR-induced photosynthesis and stress tolerance involves a H_2_O_2_-mediated increase in the GSH/GSSG ratio, which can positively regulate the synthesis and activation of redox-sensitive enzymes involved in carbon fixation (Jiang *et al.*, 2012). Accordingly, BRs play an important role in the defence and stress responses and in the maintenance of a reduced cellular redox state in plants. Taken together, these studies support the involvement of BR in plant responses to various stresses.

### Crosstalk between BRs and ABA during H_2_O_2_ generation, defence responses, and stress tolerance

In addition to BRs, ABA is well established as having positive effects on stress tolerance, and both BRs and ABA can trigger H_2_O_2_ production by NADPH oxidase in the apoplast (Pei *et al.*, 2000; Kwak *et al.*, 2003; Hu *et al.*, 2005; Xia *et al.*, 2009; Cui *et al.*, 2011). In the present study, we observed that both ABA and EBR increased the levels of *RBOH1* transcripts in wild-type and BR-deficient mutant plants (Fig. 2a). However, EBR increased *RBOH1* transcript levels at early time points but not at late time points after treatment in the ABA-deficient *not* mutant plants (Fig. 2b), which was consistent with the changes in H_2_O_2_ accumulation and antioxidative response (Figs 3, 7 and 8). Intriguingly, although ABA completely rescued the BR-deficient mutant in terms of stress tolerance, EBR rescued the stress tolerance of ABA-deficient mutant only at 3h and not at 24h after application (Fig. 1). The expression analysis of defence-related genes also provided further evidence that EBR could only transiently rescue the ABA-deficient mutant in the early stages after treatment (Fig. 7). These results suggest that the inability of EBR to induce prolonged stress tolerance in the *not* mutant plants was associated with the lack of sustained H_2_O_2_ accumulation in the late stage.

There are conflicting results regarding the relationship between BRs and ABA in stress tolerance. Several *in vivo* studies using chemical inhibitors have demonstrated that inhibition of ABA with a biosynthesis inhibitor compromises BR activity during the stress response, other studies have shown that EBR significantly improves the tolerance of an ABA-deficient mutant and that the effect of BRs on stress tolerance was ABA-independent (Divi *et al.*, 2010; Liu *et al.*, 2011; Zhang *et al.*, 2011). These discrepancies could be explained by time-dependent changes in the *RBOH1* transcript level, H_2_O_2_ accumulation, and defence responses (Figs 2 and 3). When plants were exposed to a continuous supply of BRs, *RBOH1* induction and H_2_O_2_ production occurred continuously, without the aid of ABA. Taken together, these results strongly suggest that ABA biosynthesis plays an important role in sustained stress tolerance in BR-induced pathways in plants.

### Different sources of H_2_O_2_ in EBR- and ABA-induced stress tolerance

Similar to ABA, BRs induce H_2_O_2_ accumulation by inducing/activating *RBOH1*-NADPH oxidase in the apoplast (Hu *et al.*, 2005; Xia *et al.*, 2009; Cui *et al.*, 2011). Thus, a critical question is whether BR and ABA induce H_2_O_2_ production through the same pathway and whether BR-triggered H_2_O_2_ production is dependent on ABA. In the present study, we found that BRs could transiently induce H_2_O_2_ accumulation, which was independent of ABA biosynthesis; however, sustained induction was ABA dependent (Fig. 3). Interestingly, BR only induced H_2_O_2_ accumulation in the apoplast; however, ABA induced H_2_O_2_ accumulation not only in the apoplast but also in the chloroplasts (Figs 3 and S2). Apparently, BRs directly induced increases in *RBOH1* transcripts, and *RBOH1* silencing led to decreased H_2_O_2_ accumulation and compromised the effects of EBR on H_2_O_2_ accumulation and stress tolerance (Figs 4 and 5). Importantly, unlike in the wild-type and *not* plants, EBR was unable to induce tolerance to PQ in *RBOH1*-silenced plants (Fig. 5), even when applied at relatively high concentrations (up to 1000nM). Therefore, our results showed that EBR-induced tolerance is dependent on H_2_O_2_ production in the apoplast by NADPH oxidase. In contrast, ABA was able to induce PQ tolerance at a wide range of concentrations (up to 50–100 µM; Fig. 5d) in *RBOH1*-silenced plants, and this ABA-induced PQ tolerance was compromised when DMTU was co-applied (Fig. 5a). These observations indicate that the induction of PQ tolerance by ABA at moderate concentrations is not dependent on *RBOH1* because ABA could also induce H_2_O_2_ accumulation in the chloroplasts independent of *RBOH1*, which apparently plays an important role in ABA-induced tolerance. Therefore, ABA induces PQ tolerance by triggering H_2_O_2_ accumulation in the apoplast and chloroplasts.

### Involvement of ROS in BR-induced ABA biosynthesis and prolonged stress tolerance

It remains to be clarified whether BR can induce ABA biosynthesis and, if so, whether ROS is required for this process. Several studies have shown that BR-mediated tolerance to high temperature, *Phytophthora infestans* infection, and water stress is associated with enhanced ABA accumulation (Krishna, 2003; Kurepin *et al.*, 2008; Zhang *et al.*, 2011). However, other studies have shown that pre-treatment with BR decreases stress-induced ABA accumulation (Shakirova and Bezrukova, 1998; Avalbaev *et al.*, 2010). It is worth noting that all of these results were obtained in plants after long stresses and there is no genetic evidence to support the involvement of BRs in ABA biosynthesis. In the present study, we found that ABA levels were decreased in the BR biosynthetic mutant but could be increased by exogenous EBR application (Fig. 6a). Interestingly, the ABA content was reduced, and exogenous BR failed to increase ABA accumulation in the *RBOH1*-silenced plants, suggesting a role for H_2_O_2_ in EBR-induced ABA biosynthesis (Fig. 6b). Similarly, Zhang *et al.* (2011) reported that NO was involved in BR-induced ABA biosynthesis and similar to ROS, NO is a downstream signalling molecule of H_2_O_2_ in BR signalling (Cui *et al.*, 2011). Further evidence for the role of H_2_O_2_ in ABA biosynthesis is based on the increase in ABA biosynthesis in plants exposed to PQ (Fig. 6b), which generates ROS in the chloroplasts during exposure to light. Similarly, Galvez-Valdivieso *et al.* (2009) found that exposure to high light stress induced the accumulation of both ROS and ABA in *Arabidopsis*. However, the EBR-induced increase in ABA observed in our study was much less than that observed in other studies (Liu *et al.*, 2011; Zhang *et al.*, 2011). In our study, the intact plants received only a single EBR treatment, whereas in other studies, *in vivo* shoots in BR solution or cell-culture suspensions were continuously exposed to EBR (Liu *et al.*, 2011; Zhang *et al.*, 2011), which may lead to the continuous uptake of BRs and activation of *RBOH1*. Most recently, we found that a high level of BR could induce a prolonged increase in ROS, which formed a positive amplification loop with ABA signaling in stomatal closure (Xia *et al.*, 2014). Consequently, higher levels of ROS may result in ABA accumulation.

In summary, we present genetic and molecular evidences for the dynamic interplay between BR- and ABA-induced H_2_O_2_ in tomato stress tolerances. Following the perception of a BR signal, *RBOH1*-NADPH oxidase is activated to produce H_2_O_2_. Increased ROS can then trigger increased ABA biosynthesis, which in turn causes a further increase in H_2_O_2_ production leading to prolonged stress tolerance. At concentrations effective for the induction of stress tolerance, ABA induced H_2_O_2_ production from two distinct sources (i.e. the apoplastic and chloroplastic compartments) (Fig. 9).

## Supplementary data

Supplementary data are available at *JXB* online.

Supplementary Fig. S1. Effects of BRs and ABA levels on heat-shock and photo-oxidative stress tolerance in BR- and ABA-deficient plants.

Supplementary Fig. S2. The roles of BR and ABA in regulation of H_2_O_2_ accumulation and PQ tolerance.

Supplementary Fig. S3. The *in situ* detection of H_2_O_2_ in leaves.

Supplementary Table S1. Primers used for real-time RT-PCR assays.

Supplementary Data

## Abbreviations:

ABA: abscisic acid
AC: Ailsa Craig
APX: ascorbate peroxidase
AsA: reduced ascorbate
BR: brassinosteroid
CAT: catalase
CR: Condine Red
DAB: 3,3′-diaminobenzidine
DHA: oxidized ascorbate
DMTU: dimethylthiourea
DPI: diphenylene iodonium
EBR: 24-epibrassinolide
GR: glutathione reductase
GSH: reduced glutathione
GSSG: oxidized glutathione
NO: nitric oxide
PPFD: photosynthetic photon flux density
PQ: paraquat
qRT-PCR: quantitative real-time PCR
ROS: reactive oxygen species
SOD: superoxide dismutase
TRV: tobacco rattle virus
VIGS: virus-induced gene silencing.
