# Supplementary material for: H2O2 mediates the crosstalk of brassinosteroid and abscisic acid in tomato responses to heat and oxidative stresses
Source: J Exp Bot. 2014 Jun 4;65(15):4371–83. doi: 10.1093/jxb/eru217 (PMC4112640; doi:10.1093/jxb/eru217)
Supplement: Supplementary Data [file supp_65_15_4371__index.html]

H2O2 mediates the crosstalk of brassinosteroid and abscisic acid in tomato responses to heat and oxidative stresses — H2O2 mediates the crosstalk of brassinosteroid and abscisic acid in tomato responses to heat and oxidative stresses — Supplementary Data 

# H2O2 mediates the crosstalk of brassinosteroid and abscisic acid in tomato responses to heat and oxidative stresses

## Supplementary Data

Data files

**Files in this Data Supplement:**

- Supplementary Data - Supplementary Data
