# Supplementary material for: H2O2 mediates the crosstalk of brassinosteroid and abscisic acid in tomato responses to heat and oxidative stresses
Source: J Exp Bot. 2014 Jun 4;65(15):4371–83. doi: 10.1093/jxb/eru217 (PMC4112640; doi:10.1093/jxb/eru217)
Supplement: Supplementary Data [file supp_eru217_jexbot119008_file001.pdf]

H_2O_2 mediates the crosstalk of brassinosteroid and abscisic acid in tomato responses to heat and oxidative stresses

J Q Yu, Jie Zhou, Jian Wang, Xin Li, Zhixiang Chen, Yanhong Zhou, Kai Shi, and Xiaojian Xia

Supplementary Data

Supplementary Figure S1. Effects of BRs and ABA levels on heat shock and photooxidative stress tolerance in BR- and ABA-deficient plants. (a) Electrolyte leakage values of plants after exposure to a 6 h heat shock (42°C under 800 µmol m^{-2} s^{-1}) . (b) Electrolyte leakage values of plants after exposure to 3 h of photooxidative stress (20 µM PQ). For (a) and (b), EBR (200 nM) or ABA (50 µM) were applied at 24 h before the plants were exposed to heat shock and PQ stresses, respectively. Twelve plants were used for each treatment, and Electrolyte leakage values were determined with the full 4th leaf. The data are the means of twelve replicate plants (±SD). Means denoted by the same letter do not differ significantly at $P \leq 0.05$ according to Turkey's test. CR, Condine Red (wild-type); *d^im*, BR-deficient mutant; AC, Ailsa Craig (wild-type); *not*, *notabilis* ABA-deficient mutant.

(a) **(b)**

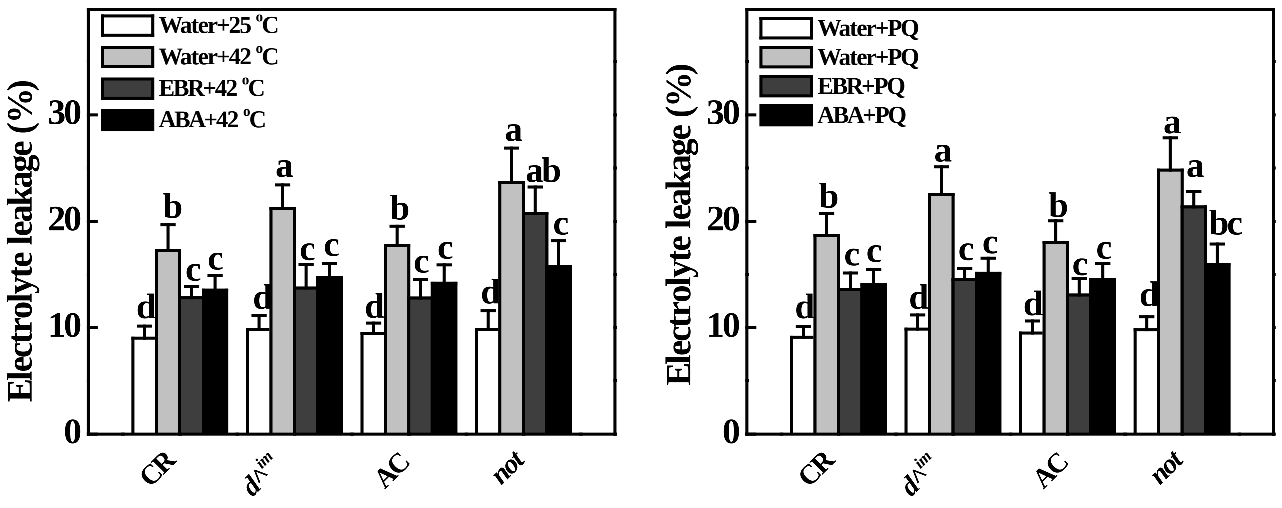

Supplementary Figure S2. The roles of BR and ABA in regulation of PQ tolerance. (a) and (b) Images and values of the maximum PSII quantum yield (Fv/Fm) of leaves after exposure to a 3 h PQ stress (20 µM) at 24 h after EBR (200 nM), or ABA (50 µM) treatments with DPI or DMTU pretreatment. DPI (50 µM) and DMTU (5 mM) were applied 12 h before the EBR or the ABA treatment. Twelve plants were used for each treatment and the picture of one representative leaf is shown. Bar = 1.0 cm.

(a)

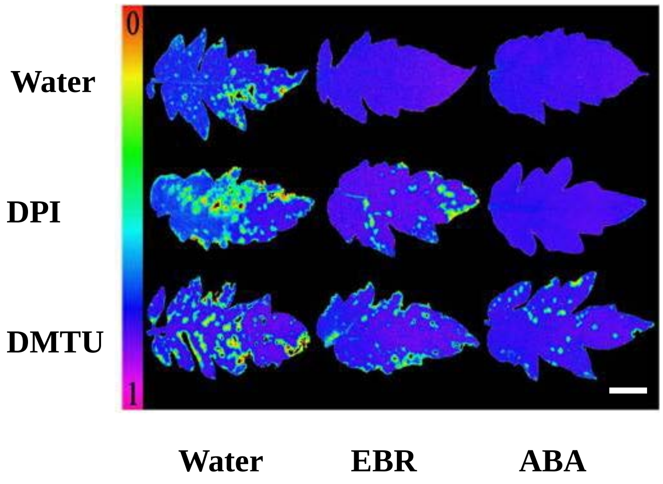

(b)

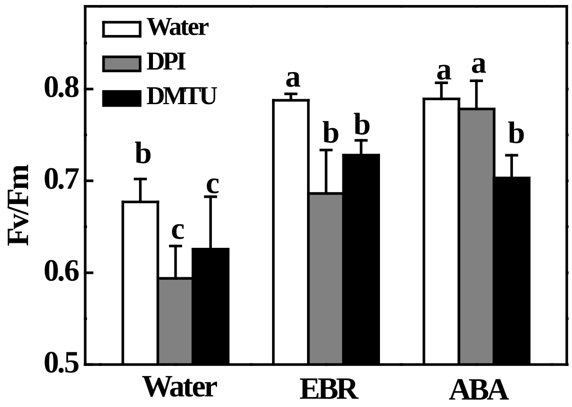

Supplementary Figure S3. *In situ* detection of H_2O_2 in leaves. Leaf samples were taken at 24 h after (200 nM), ABA (50 µM) or at 3 h after paraquat (PQ, 20 µM) or H_2O_2 (50 mM) treatment, and loaded with DAB and incubated for 6 h. DPI (50 µM) and DMTU (5 mM) were applied 12 h before the EBR or the ABA treatment. The H_2O_2 accumulation was detected by an Olympus motorized system microscope (BX61, Olympus Co., Tokyo, Japan). (a) & (b) observed at 2x magnification; (c) observed at 400x magnification.

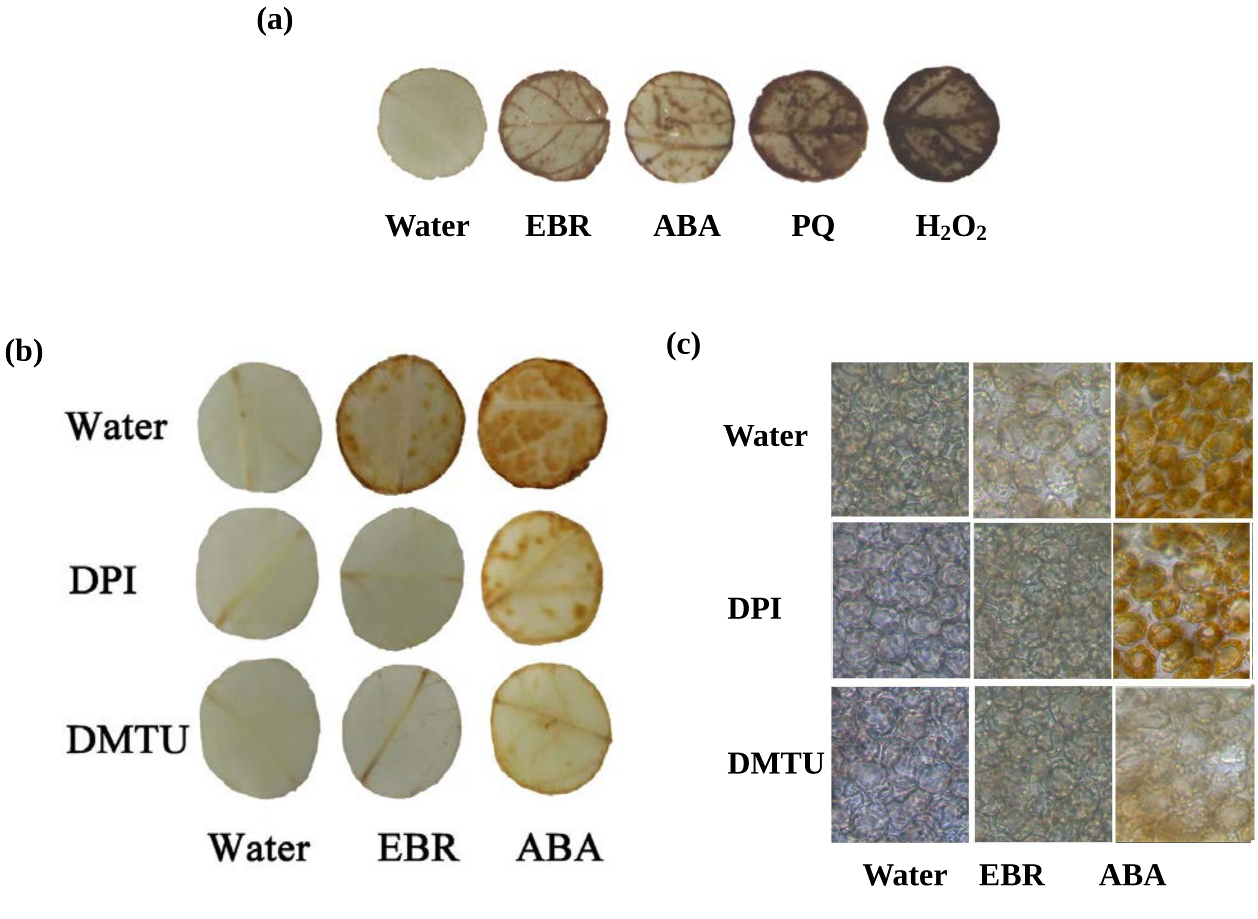

Supplementary Table S1. Primers used for real time RT-PCR assays

Gene	Accession numbers	Forward primer	Reverse primer
Rboh1	Sl08g081690	5'-GGAGCTCCAGCACAAGATTA-3'	5'-CTTGTTGCAGCACTCATGTC-3'
WRKY1	Sl07g047960	5'- CTAGTGCAGGGTCAAGGAAA-3'	5'- ACAGGACTCTTCGTCACTCG-3'
WRKY72	Sl02g067430	5'- AAGCAGGTTCAAAGATGTGC-3'	5'- CAGTTACTTGTGGGTTTGGG-3'
MAPK1	Sl12g019460	5'-TGCACCTCCGGTCAACAA-3'	5'-GGCAGTGCTCCTCAGATAAA-3'
HSP70	NM001246851	5'- CAAGCTGAAAGAGCTCAAGG-3'	5'- CTGTCCCAGCTGCATTACTT-3'
Cu/Zn-SOD	Sl11g066390	5'- GGCCAATCTTTGACCCTTTA -3'	5'- AGTCCAGGAGCAAGTCCAGT -3'
cAPX	Sl06g005160	5'- TCTGAATTGGGATTTGCTGA -3'	5'- CGTCTAACGTAGCTGCCAAA -3'
GR1	Sl09g091840	5'- TTGGTGGAACGTGTGTTCTT -3'	5'- TCTCATTCACTTCCCATCCA -3'
CAT1	Sl12g094620	5'-TGATCGCGAGAAGATACCTG-3'	5'-CTTCCACGTTCATGGACAAC-3'
Actin	Sl11g005330	5'- TGTCCCTATTTACGAGGGTTATGC -3'	5'- CAGTTAAATCACGACCAGCAAGAT -3'
